# PSMIX: an R package for population structure inference via maximum likelihood method

**DOI:** 10.1186/1471-2105-7-317

**Published:** 2006-06-22

**Authors:** Baolin Wu, Nianjun Liu, Hongyu Zhao

**Affiliations:** 1 Division of Biostatistics, School of Public Health, University of Minnesota, Minneapolis, MN, USA; 2 Department of Biostatistics, University of Alabama at Birmingham, Birmingham, AL, USA; 3 Department of Epidemiology and Public Health, Yale University School of Medicine, New Haven, CT, Yale University School of Medicine, New Haven, CT, USA; 4 Department of Genetics, Yale University School of Medicine, New Haven, CT, Yale University School of Medicine, New Haven, CT, USA

## Abstract

**Background:**

Inference of population stratification and individual admixture from genetic markers is an integrative part of a study in diverse situations, such as association mapping and evolutionary studies. Bayesian methods have been proposed for population stratification and admixture inference using multilocus genotypes and widely used in practice. However, these Bayesian methods demand intensive computation resources and may run into convergence problem in Markov Chain Monte Carlo based posterior samplings.

**Results:**

We have developed PSMIX, an R package based on maximum likelihood method using expectation-maximization algorithm, for inference of population stratification and individual admixture.

**Conclusion:**

Compared with software based on Bayesian methods (e.g., STRUCTURE), PSMIX has similar accuracy, but more efficient computations.

PSMIX and its supplemental documents are freely available at .

## Background

Information about population structure, namely population stratification and admixture, is useful in a variety of situations, such as association studies of genes underlying complex traits, subspecies classification, genetic barrier detection, and evolutionary study [1-10]. For example, it is very important to identify genetic ancestry and admixture in admixture mapping [7,8]. The presence of population stratification or admixture may pose a practical nuisance as well. In association studies, case-control design is often used to identify genetic variants underlying complex traits by comparing allele frequencies between unrelated individuals that are affected and those unaffected. However, the presence of population stratification or admixture in the sample can lead to spurious associations between a candidate marker and a phenotype [5,10,11]. In forensic studies, the identification of reference groups is central but becomes difficult when there exists population stratification [12,13]. In the estimation of the magnitude of inbreeding, it is useful to distinguish between the causes for the excess homozygosity which might be consanguineous mating or population substructure, or an artifact due to factors like null alleles [14]. In all these situations, identifying population stratification or admixture has been an important component.

Population structure can be identified based on visible characters such as language, culture, physical appearance, and geographic region. But this can be subjective and may bear no relevance to genetics. Evanno et al. [15] gave a good example by mentioning migratory bats which can be found thousands of kilometers apart but from the same breeding roost in winter [16]. Statistical methods have been proposed to infer population stratification and individual admixture using multilocus genotype data [1,2,17-31]. Different methods use different statistical tools and population genetic assumptions. Pritchard et al. [2] introduced a model-based clustering method to infer population structure and assign individuals to populations using multilocus genotype data. They used Bayesian formulation and generated the posterior distributions using a Markov Chain Monte Carlo (MCMC) method based on Gibbs sampling. Their main modeling assumptions are Hardy-Weinberg equilibrium (HWE) within populations and linkage equilibrium (LE) between markers within each population. Falush et al. [21] extended the method to allow for loose linkage between loci. The method of Corander et al. [17,18] uses multilocus molecular markers and geographical information provided by the sampling design. Unlike the methods of Pritchard et al. [2] and Falush et al. [21], the methods of both Dawson and Belkhir [19] and Corander et al. [17,18] can directly estimate the number of (sub)populations and assign individuals to the (sub)populations. The main difference between the two approaches is the parametric assumption of the number of populations [17-19,32]. Corander et al. [18] considered the geographical sampling design of the individuals and set the maximum number of populations allowed to be the number of locations sampled, whereas for Dawson and Belkhir [19], it is the total number of individuals. Corander et al. [17] generalized the approach of Corander et al. [18] and it became more similar to the approach of Dawson and Belkhir [19] in terms of model assumptions and some technical details, especially when the data is specified for individual level analysis. Guillot et al. [23,24] used spatial statistical models employing both landscape ecology and population genetics information, which is especially useful in situations of young populations exhibiting low genetic differentiation [23,33]. Excoffier et al. [20] applied approximate Bayesian computation method to the estimation of all the parameters of an explicit admixture model. Their method can easily deal with complex mutation models and partially linked loci and is superior when the admixture is more ancient [20]. The majority of the methods for population structure inference are Bayesian approaches [1,2,17-26] with few exceptions such as Tang et al. [30], Satten et al. [29], Wang [31], and Purcell and Sham [28]. Meanwhile, several methods have been proposed for the assignment of individuals to populations [34-36]. As for computer programs available based on existing methods, the majority are also based on Bayesian MCMC methods, such as STRUCTURE [2,21], GENELAND [24], BAPS/BAPS 2 [17,18], and ADMIXMAP [25,37,38], with the exception of L-POP[28] which is based on latent class analysis. Table 1 summarizes some of the commonly used software for population structure inference. STRUCTURE is the most commonly used program for population structure inference which has been used both on humans [4,13,39] and other species [3,40-42] (at the time this article is written, the paper of Pritchard et al. [2], where the method of STRUCTURE was originally proposed, has been cited about 760 times). We choose to compare the performance of our package with STRUCTURE and L-POP (the representative of the frequentist methods).

## Implementation

We have developed an efficient R package, named PSMIX (Population Structure inference via MIXture model), for population stratification and individual admixture inference. Since R can be slow when computation is intensive, we implemented the expectation-maximization (EM) algorithm [43] using C programming language. PSMIX is mainly based on the methods proposed in Tang et al. [30] and Liu et al. [27]. Three models (described in section 2.2, 2.3, and 2.4, respectively) are discussed in full detail in [27]. The second one is equivalent to the model proposed in Tang et al. [30]. The first model is a special case of the second one. In Tang et al. the method itself has been fully assessed by simulation studies [30].

## Results

We used two real datasets from Rosenberg et al. [4] and one simulated dataset from Tang et al. [30] to demonstrate the functionality of PSMIX. One real dataset contains two American populations, Pima and Surui with 25 and 21 individuals, respectively; the other contains two European populations, Sardinian and Russian with 28 and 25 individuals, respectively. The simulated data set contains 50 individuals from each of the two ancestral populations, and 200 individuals from the admixed population. The true individual admixture values of the admixed individuals are also available.

To evaluate the efficiency of PSMIX, we randomly selected 100 markers from the Pima-Surui dataset with no missing values and tried the four models available in STRUCTURE2.0. Burnin length and number of MCMC replications after burnin were both set to be 10,000 in the analyses. The time needed for each run of STRUCTURE2.0 increased almost linearly with the increase of number of clusters. On our PC with Pentium III 500 MHZ CPU and 384 MB SDRAM, when K = 2, about two and a half minutes were needed for each run of STRUCTURE2.0. For all PSMIX runs, we set the stopping criterion to be that the parameter difference <10^-6 ^between consecutive iterations, or 10,000 steps, whichever was reached first. For the same Pima-Surui data with 100 markers, each run of PSMIX needed about 6 seconds.

**Figure 1:**
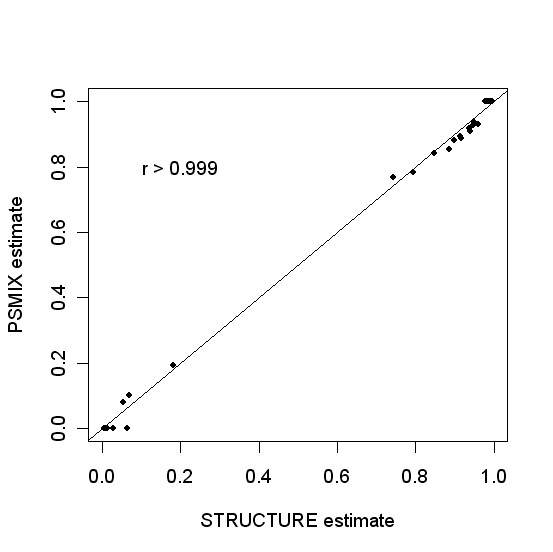
Comparison of estimates of individual admixture of STRUCTURE (x-axis) and PSMIX (y-axis) for the data of Pima and Surui from Rosenberg et al. (2002). Only the first 50 markers were used.

**Figure 2:**
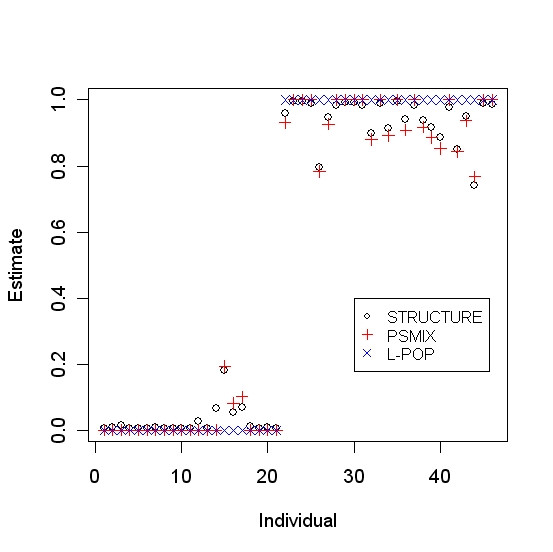
Estimates of STRUCTURE, PSMIX and L-POP for the data of Pima and Surui (the first 21 samples are Surui) from Rosenberg et al. (2002). Only the first 50 markers were used.

To evaluate the accuracy of PSMIX, we compared the results of PSMIX with those of STRUCTURE. Figure 1 gives a sample run for the Pima-Surui dataset using only the first 50 markers. For the Pima-Surui dataset using the first 50 markers from the original data, the correlation coefficient between the results of PSMIX and STRUCTURE was greater than 0.999. For the Sardinian-Russian dataset, when all 377 markers were used, both STRUCTURE 2.0 (use independent allele frequencies among populations with admixture model) and PSMIX had one individual misclustered. The correlation coefficient between the results was 0.906. The two methods produced very similar results. This is consistent with the findings in Tang et al. [30]. Figure 2 shows the results of STRUCTURE, L-POP, and PSMIX. We can see that the results of PSMIX are much closer to those of STRUCTURE.

**Figure 3:**
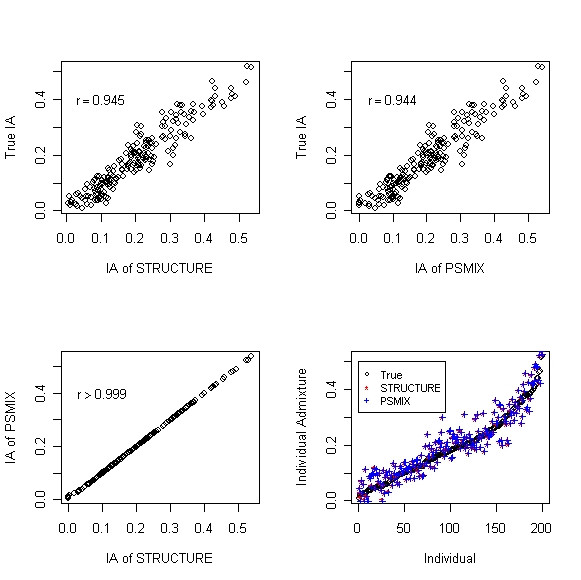
Estimates of STRUCTURE and PSMIX for the simulated data.

To evaluate the performance of PSMIX, we also used a simulated data set exhibiting population admixture. From Figure 3 we can see the PSMIX performs pretty well and the results are almost identical to those from STRUCTURE.

## Discussion

We have implemented a likelihood based method of population structure inference into an efficient R package, PSMIX. PSMIX can be used in population genetics and disease gene mapping, wherever population stratification or individual admixture is needed to be estimated from genetic markers. Compared with other available similar programs, PSMIX has several advantages. First, it is computationally efficient and provides similar accuracy under realistic situations (Tang, et al. [30] and Liu et al., Technical Report [27]). And thus the confidence intervals of the estimates can be constructed via resampling methods, e.g., the bootstrap method [30]. Second, as shown in Tang et al. [30], it performs a little better (compared with STRUCTURE) under some conditions involving a small number of ancestors and markers. We note that L-POP is also computationally efficient. However, it is not clear if L-POP can perform better under such conditions. Third, it is very flexible. It is likelihood based and can be easily incorporated into study designs, such as marker choice [30]. The program is implemented as a public R package and can be easily extended and incorporated into other packages. This is an advantage of PSMIX over STRUCTURE and L-POP, which has only executable programs.

We would like to note that the examples used in this work are mainly for the purpose of demonstrating the R package, not for the purpose of the assessing the underlying method. Please refer to Tang et al. [30] for a detailed assessment of the methodology.

In our simulation and application to real data, PSMIX and STRUCTURE gave very similar results. This is not surprising because estimating parameters via maximum likelihood and maximum a posterior with flat prior is formally strictly similar, where PSMIX belongs to the former and STRUCTURE belongs to the latter.

Many studies have been performed to assess the ability of STRUCTURE in assigning individuals to their populations of origin using either real data or simulated data [3,44-47]. However, very limited studies have been performed to assess the ability of STRUCTURE in detecting the number of populations. Recently, Evanno et al. [15] performed a systematic study on this issue using simulations. They simulated amplified fragment length polymorphism (AFLP) and microsatellite genetic data under three population structure models: the island model, a contact zone, and a hierarchical island model [15]. Their major finding is that the "log probability of data", an ad hoc criterion suggested by Pritchard et al. [2] for detecting the number of populations, does not provide a correct estimation of the number of populations most of the time [15]. However, they found that another ad hoc statistic, which is based on the rate of change in the log probability of the data between successive numbers of populations, can accurately detect the uppermost hierarchical level of structure [15]. They also found some other factors that can affect the detection of the number of populations [15]. These findings are important and useful in that with the increasing usage of STRUCTURE, they provide guidance on how to use STRUCTURE to detect the number of populations. However, PSMIX does not directly detect the number of populations in this version. Due to its computation efficiency, model selection methods such as Akaike information criterion (AIC) [28,48], Bayesian information criterion (BIC) [49], and even more general, penalized likelihood based methods [50,51] can be used for this purpose. The findings of Evanno et al. [15] may be incorporated into PSMIX as well. This is one of our future works.

EM approach and Bayesian MCMC approach have their own advantages and disadvantages. They both can trap in local modes, although theoretically speaking, Bayesian MCMC approach can converge to the true value eventually, maybe after an unrealistic long time. However, the Bayesian MCMC approach, in addition, has the label switching problem. Two authors (Stephens and Donnelly) of the paper where the method of STRUCTURE was proposed [2] mentioned in other papers [52,53] on methods to deal with this problem. Although this issue is believed to be well addressed by STRUCTURE, it does make the Bayesian MCMC approach more complicated. However, this topic is beyond the scope of this work. From the users' point of view, they only see the computation efficiency and stability of the methods.

We think that it may be necessary to explicitly explain some details about the models mentioned in this work. First, the orientation of Tang et al. [30] is different from that of STRUCTURE, L-POP, and Liu et al. [27]. The goal of the former was to estimate individual admixture for the admixed individuals. The original focus of the latter was to "identify discrete clusters roughly corresponding to subpopulations" [30]. STRUCTURE, L-POP, and Liu et al. [27] use methods for clustering, although they "can also be applied to an admixture model" [30]. So initially, Tang et al. [30] faced a population (the "admixed group" in their paper) that is currently in Hardy-Weinberg equilibrium, but was created as the result of admixture at some point in the past. However, as emphasized in Tang et al. [30], the problem may not be identifiable without the inclusion of pseudo-ancestors who are proxies of the true pure ancestry [25]. Here the nonidentifiablity issue is related to the problem, and by no means pertains to the method. In other words, the nonidentifiablity issue exists and has nothing to do with the statistical methods to be used, if pseudo-ancestors are not included. Therefore, the actual data Tang et al. [30] dealt with consist of "I^0 ^individuals from the admixed group, as well as I^K ^subjects from each of the K ancestral populations" [30], that is, a stratified "pooled" population. So the actual data all these methods deal with are the same in the sense that the data consist of stratified populations within which Hardy-Weinberg equilibrium holds. One major difference is that Tang et al. [30] only focus on the individual admixture of the people in the admixed population (their original population). Facing the same data, the method in Tang et al. [30] is for clustering as well, in spirit. They included pseudo-ancestors and used clustering method in order to estimate individual admixture. In other words, all the aforementioned methods are for population stratification, and can be applied to estimate individual admixture. Thus the comparisons made in this work are appropriate. We also want to emphasize here the importance of inclusion of ancestral populations or their surrogates when individual admixture is needed; otherwise the problem may not be identifiable no matter what method to use.

## Conclusion

In summary, we have implemented a new, likelihood based method for inference of population stratification and individual admixture which is available as a public R package. Although the package has several advantages over its peers, we strongly suggest that the users use different software in their analysis. If the results from these software are consistent; this may provide more support for the results; if the results are not consistent, further investigation is needed. A potential limitation is the assumption of independence among markers behind PSMIX, which will be addressed in future versions of PSMIX.

## Availability and requirements

**Project name: **PSMIX

**Project home page: **

**Operating system(s): **MS Windows, Linux, Mac

**Programming language: **C, R

**Other requirements: **R 2.0 or higher

**License: **GPL

**Any restrictions to use by non-academics: **none

## Authors' contributions

BW participated in the design of the study, implemented PSMIX, and helped to draft the manuscript; NL participated in the design of the study, performed the analysis, and drafted the manuscript; HZ conceived the study, participated in its design and helped to draft the manuscript. All authors read and approved the final manuscript.

## Figures and Tables

**Table 1:** Commonly used software for population structure inference

Software	STRUCTURE	GENELAND	BAPS/BAPS2	ADMIXMAP	PARTITION	L-POP	PSMIX
Method	Bayesian MCMC	Bayesian MCMC	Bayesian, MCMC is used when the number of populations ≥ 9	Bayesian MCMC	Bayesian MCMC	Latent class analysis, EM	Clustering analysis, EM

Features	Population structure inference	Process geo-referenced individual multilocus genetic data for population structure inference	Population structure inference. Use geographical sampling design of the individuals	Mainly for analysis of datasets that consist of trait measurements and genotype data on a sample of individuals from an admixed or stratified population	Population structure inference	Population structure inference	Population structure inference

Assumptions	HWE and LE between loci	HWE, LE between loci, and spatial distribution of sub-populations	HWE and LE between loci	Ancestry state is the same at all loci within a compound locus on any gamete. Mating is not assortative for admixture in the population from which the parental gametes were drawn	HWE and LE between loci. The underlying population genetic model is appropriate for out-crossing diploid organisms.	HWE and LE between loci	HWE and LE between loci

Input parameters	Parameters for running MCMC, parameters for ancestry model and allele frequency model, and the number of populations	In addition to genetic and spatial data, the user must provide parameters for the maximum number of populations, the way geographical information is handled and the allele frequency model	When MCMC is used, need parameters for running MCMC	Parameters for running MCMC, allele frequencies (number of population is specified here), and mating model. Disease information (outcome variable is suggested even if focus on population structure). Parameters for tests and output	Parameters for running MCMC, maximum number of populations, prior parameter for allelic diversity, and prior parameter for number of populations	Number of populations, admixture option, data format options, model options, output format options, and convergence criterion	Number of populations and convergence criterion

Output	One file for estimates and some files for plots. Main parameter estimates are inferred ancestry of individuals and estimated allele frequencies in each population	Main parameter estimates are the number of populations, population membership of each individual, maps giving the population memberships of each geographical pixel of a given size to locate genetic discontinuities between populations	Main parameter estimates are the number of populations and population membership of each individual	Individual/gamete level admixture variables. Ancestry-specific allele or haplotype frequencies. Results for association analysis and model parameters	The output file contains a list of the parameter settings followed by the sequence of observations of the Markov chain. A companion program PartitionView is provided to obtain useful information from the PARTITION output file.	Main outputs are estimates of allele frequencies, posterior class probabilities, and class-specific allele frequencies	Main parameter estimates are inferred ancestry of individuals

Advantages	Easy to use. Once number of populations is given, the estimates are accurate	Easy to use. Flexible to extend. Can work with or without spatial information. Can estimate number of populations	Easy to use. Provide good estimate for number of populations. When geographical sampling information is applicable, can improve the statistical power to detect clusters in the data	In addition to population structure inference, can perform association analysis on structured populations. Can deal with tightly linked loci using haplotypes	Easy to use. Can estimate number of populations and calculate a Bayes factor in support of a single source population against the alternative of more than one source population.	Computationally efficient	Easy to use. Computationally efficient. Flexible to extend

Limitations	Computationally intensive. Can detect number of populations but does not work well.^#^	Does not handle admixture. Computationally intensive, especially when "Falush" is used as allele frequency model, or number of populations needs to be estimated.	Very memory intensive. When MCMC is used, becomes relatively computationally intensive. Only provides membership partition, does not handle admixture	Difficult to use. Computationally intensive. Does not estimate number of populations *	Computationally intensive, especially when number of populations needs to be estimated	Parameter configuration is difficult to use, works OK for discrete populations but not for admixed populations. Does not estimate number of populations	Does not estimate number of populations

Platforms	Windows, Unix/Linux	Windows/Linux/Mac (R package)	Windows	Windows, Unix/Linux. R statistical package is required	Windows	Windows (DOS), Unix/Linux	Windows/Linux/Mac (R package)

References	Pritchard et al. (2000), Falush et al. (2003)	Guillot et al. (2005)	Corander et al. (2003, 2004)	McKeigue et al. (2000), Hoggart et al. (2003, 2004)	Dawson and Belkhir (2001)	Purcell and Sham (2004)	Tang et al. (2005), Liu et al. (2005)

URL							

^# ^With the findings of Evanno et al. (2005), STRUCTURE's ability to detect number of populations should be improved greatly.

* Only focus on the function of population structure inference.
